# Navigating the Challenges of *Stenotrophomonas maltophilia* Infections in Chronic Kidney Disease: A Case Report

**DOI:** 10.1002/ccr3.72483

**Published:** 2026-04-08

**Authors:** Joydip Chowdhury, Prince Shah‐Riar, Druba Sheesh Debnath, Shahida Afnan, Muniza Mobin Maha

**Affiliations:** ^1^ Comilla Medical College Comilla Bangladesh; ^2^ DHR Health, Edinburg, Texas University Houston Houston Texas USA; ^3^ Shaheed M. Monsur Ali Medical College Dhaka Bangladesh; ^4^ Sylhet Women's Medical College Sylhet Bangladesh

**Keywords:** antimicrobial resistance, chronic kidney disease, multidrug resistant organism, nosocomial pneumonia, *Stenotrophomonas maltophilia*

## Abstract

*S. maltophilia* is a multidrug‐resistant nosocomial pathogen that poses significant challenges in CKD patients. Antibiotic selection in CKD is complicated by nephrotoxicity concerns and limited clinical data. Combination therapy and novel agents like cefiderocol and CZA‐ATM are emerging alternatives but require further validation.

## 1 Introduction

*Stenotrophomonas maltophilia* is an emerging Gram‐negative pathogen associated with significant morbidity in hospitalized patients, particularly those with CKD [1]. The bacterium is known for its intrinsic resistance to multiple antibiotics, leading to limited treatment options and increased mortality rates, which can reach up to 37.5% [2]. The World Health Organization (WHO) has highlighted *S. maltophilia* as a priority pathogen due to its resistance profile and its role in hospital‐acquired infections, particularly ventilator‐associated pneumonia and bacteremia [3].

## 2 Case Presentation

A 100‐year‐old male with a history of heart failure with reduced ejection fraction (EF‐20%), CKD stage 4, and CAD (post‐coronary artery bypass grafting in 1997) was brought to the emergency department with severe respiratory distress. He had recently been discharged following a two‐week hospitalization for an ischemic stroke, which resulted in significant neurological deficits and dysphagia requiring nasogastric (NG) tube placement.

Upon arrival, the patient was intubated due to respiratory failure. Imaging revealed bilateral acute infarcts with hemorrhagic transformation, pleural effusions, and pulmonary edema. Empiric broad‐spectrum antibiotic therapy with vancomycin and piperacillin‐tazobactam was initiated. Neurological evaluation confirmed evolving infarcts, and CT angiography revealed left internal carotid artery occlusion.

On hospital day 5, sputum cultures identified *S. maltophilia* , prompting a switch to intravenous levofloxacin, as TMP‐SMX was contraindicated due to CKD. Metronidazole was added for anaerobic coverage. Microscopic details of the Gram stain and leukocyte count from the respiratory aspirate were not fully documented in the microbiology report. However, sputum culture subsequently identified *Stenotrophomonas maltophilia* , and laboratory findings showed leukocytosis (WBC 18.2 × 10^9^/L) with neutrophil predominance, supporting an active infectious process. Despite targeted therapy, the patient remained ventilator‐dependent, leading to tracheostomy and percutaneous endoscopic gastrostomy (PEG) placement for prolonged respiratory and nutritional support.

The hospital course was further complicated by CKD‐related anemia requiring transfusion and colitis diagnosed via abdominal imaging. The infectious disease, nephrology, and neurology teams collaborated closely in his management. Despite aggressive supportive care, the patient's condition remained critical, and discussions regarding long‐term care planning were initiated with the family.

## 3 Differential Diagnosis

The differential diagnosis includes various gram‐negative and multidrug‐resistant pathogens, each requiring careful evaluation as it significantly impacts patient management.

## 4 Outcome and Follow‐Up

The 100‐year‐old patient with CKD stayed on a ventilator despite antibiotics, with no signs of improvement. A tracheostomy for breathing and a percutaneous endoscopic gastrostomy (PEG) tube for feeding while discussing long‐term care options with the family. The goal was to manage the infection, keep the patient comfortable, and arrange for the best possible care.

## 5 Discussion

This case highlights the complexities of treating *S. maltophilia* infections in elderly patients with CKD [4]. CKD patients have altered immune responses, increased susceptibility to infections, and require careful antibiotic selection due to nephrotoxicity risks. The absence of standardized antimicrobial dosing guidelines for CKD patients without renal replacement therapy complicates clinical decision‐making [5, 6].

TMP‐SMX remains the preferred first‐line treatment for *S. maltophilia* [6]; however, alternatives such as levofloxacin and ceftazidime are commonly used in patients with renal impairment [7]. The lack of standardized susceptibility breakpoints further complicates antibiotic selection [8].

Emerging data suggest that combination therapy—including TMP‐SMX with minocycline or levofloxacin—may be more effective than monotherapy. Novel agents such as cefiderocol and CZA‐ATM offer promising alternatives, particularly for resistant strains, though long‐term clinical outcome data remain limited [9, 10, 11].

In CKD patients, the pharmacokinetics of antimicrobials are significantly altered, necessitating dose adjustments to prevent toxicity while maintaining efficacy. Multidisciplinary collaboration between nephrologists, infectious disease specialists, and intensivists is crucial in optimizing treatment outcomes. Future research should focus on long‐term follow‐up studies and real‐world efficacy data for novel antimicrobial regimens in this population [9, 10, 11, 12].

## Author Contributions

**Joydip Chowdhury:** conceptualization, investigation, writing – original draft. **Prince Shah‐Riar:** conceptualization, investigation, supervision, writing – original draft, writing – review and editing. **Druba Sheesh Debnath:** conceptualization, investigation, writing – original draft. **Shahida Afnan:** writing – review and editing. **Muniza Mobin Maha:** writing – review and editing.

## Funding

The authors have nothing to report.

## Consent

Written informed consent for publication of their details was obtained from the patient.

## Conflicts of Interest

The authors declare no conflicts of interest.

## Data Availability

Data sharing not applicable to this article as no datasets were generated or analysed during the current study.
